# Increased Risk of Non-Fatal Myocardial Infarction Following Testosterone Therapy Prescription in Men

**DOI:** 10.1371/journal.pone.0085805

**Published:** 2014-01-29

**Authors:** William D. Finkle, Sander Greenland, Gregory K. Ridgeway, John L. Adams, Melissa A. Frasco, Michael B. Cook, Joseph F. Fraumeni, Robert N. Hoover

**Affiliations:** 1 Consolidated Research, Inc., Los Angeles, California, United States of America; 2 Department of Epidemiology and Department of Statistics, University of California, Los Angeles, California, United States of America; 3 Division of Cancer Epidemiology and Genetics**,** National Cancer Institute, Bethesda, Maryland, United States of America; College of Pharmacy, University of Florida, United States of America

## Abstract

**Background:**

An association between testosterone therapy (TT) and cardiovascular disease has been reported and TT use is increasing rapidly.

**Methods:**

We conducted a cohort study of the risk of acute non-fatal myocardial infarction (MI) following an initial TT prescription (N = 55,593) in a large health-care database. We compared the incidence rate of MI in the 90 days following the initial prescription (post-prescription interval) with the rate in the one year prior to the initial prescription (pre-prescription interval) (post/pre). We also compared post/pre rates in a cohort of men prescribed phosphodiesterase type 5 inhibitors (PDE5I; sildenafil or tadalafil, N = 167,279), and compared TT prescription post/pre rates with the PDE5I post/pre rates, adjusting for potential confounders using doubly robust estimation.

**Results:**

In all subjects, the post/pre-prescription rate ratio (RR) for TT prescription was 1.36 (1.03, 1.81). In men aged 65 years and older, the RR was 2.19 (1.27, 3.77) for TT prescription and 1.15 (0.83, 1.59) for PDE5I, and the ratio of the rate ratios (RRR) for TT prescription relative to PDE5I was 1.90 (1.04, 3.49). The RR for TT prescription increased with age from 0.95 (0.54, 1.67) for men under age 55 years to 3.43 (1.54, 7.56) for those aged ≥75 years (p_trend_ = 0.03), while no trend was seen for PDE5I (p_trend_ = 0.18). In men under age 65 years, excess risk was confined to those with a prior history of heart disease, with RRs of 2.90 (1.49, 5.62) for TT prescription and 1.40 (0.91, 2.14) for PDE5I, and a RRR of 2.07 (1.05, 4.11).

**Discussion:**

In older men, and in younger men with pre-existing diagnosed heart disease, the risk of MI following initiation of TT prescription is substantially increased.

## Introduction

Testosterone therapy (TT) has been used in healthy older men to treat diminished extremity strength and physical function associated an age-related decline in serum testosterone. [1] Recently TT has been increasing extraordinarily rapidly, including among younger men and among those without hormone measurement, suggesting that the indications for prescription have likely markedly expanded. [2], [3] Three recent studies have raised some concerns about possible adverse cardiovascular outcomes associated with TT. In 2010 a small randomized trial of testosterone gel on muscle function in men 65 years of age or older was discontinued due to an excess of a variety of cardiovascular events in the testosterone arm. [4] This was followed by a meta-analysis of a number of a number of very small trials in predominantly older men which also suggested excess cardiovascular risk. [5] Recently, a study in the Veteran’s Administration health care system of men average age over 60, 80% of whom had documented coronary disease, reported an excess of a category of events that included death and cardiovascular disease in those receiving TT. [6] In the two studies assessing timing of the increase, it was noted to appear very soon following initiation of therapy. [4], [5] While this has raised public health concerns [2], [5], [7], significant questions remain. In all 3 studies, combined cardiovascular disease endpoints were used since individual outcomes, particularly severe events, were too few to evaluate. Perhaps because of this, or for other factors, the point estimates of risks were also divergent among the studies, with hazard ratios ranging from less than 1.3 to greater than 5.0. All of these studies also recognized the importance of evaluating risk among those men with and without preexisting heart disease, but did not have sufficient numbers of subjects to adequately assess this issue. All three studies were also predominantly of older men, and unable to address risks in younger men where the increases in prescriptions have been the most dramatic. Therefore, using a large healthcare database, we evaluated the hypotheses that TT might increase the risk of acute non-fatal myocardial infarction (MI), and that this effect might also be particularly strong in those with pre-existing cardiac disease. We also explored whether these same effects might apply to younger men as well.

## Methods

### Data Source

The Truven Health MarketScan® Commercial Claims and Encounters Database includes employees, dependents and retirees with commercial or Medicare insurance whose employers license healthcare data to Truven Health Analytics (Truven). The MarketScan data contributors include Fortune 500 employers (60 percent) and health plans covering numerous other companies and unions (40 percent). The data include diagnoses, procedures, and prescriptions for all enrollees. Enrollees in 2011 are distributed regionally within the U.S. as Northeast (24 percent), North Central (37 percent), South (20 percent), and West (19 percent). We used the data from 2006 to 2010, including patient-specific enrollment history, year of birth, gender, inpatient and outpatient diagnoses (ICD-9 codes) and treatments, and outpatient prescriptions. [8] No data were available on indications for TT prescription, race, laboratory findings, occupational, environmental, or lifestyle factors.

The data for the study were hosted in secure facilities of Consolidated Research, Inc. (CRI), as required by the Agreement between CRI (Los Angeles, California) and Truven Health Analytics (Ann Arbor, Michigan). We received IRB exemption for this study from the NIH Office of Human Subjects Research Protections (OHSRP) since the study was conducted in claims data that were anonymized or de-identified by Truven prior to release. The database is available from Truven under licensing agreements similar to ours.

### Cohort Formation

We formed cohorts from the database from men with a minimum of 22 months of continuous enrollment for analyses with post-prescription follow-up intervals of 90 days, and 25 months for analyses with post prescription follow-up intervals of 91 to 180 days. From this cohort, we selected men who filled a first prescription for any of several TT prescriptions not containing estrogen (N = 55,593) and, for a comparison population, men who filled a first prescription for phosphodiesterase type 5 inhibitors (PDE5I; sildenafil or tadalafil, N = 167,279), between January 1, 2008 and September 30, 2010. We did not have data on how much of the prescribed medication was consumed. The most common TT prescriptions were testosterone gel, testosterone micronized, testosterone cypionate, and testosterone transdermal system. We selected men receiving PDE5I prescriptions as a comparison group because some indications for prescription are similar to those for TT prescription. In addition, PDE5I is commonly prescribed to older men, does not have androgenic effects, and is not metabolized to other sex steroid hormones, such as dihydrotestosterone or estrogens. Also, while PDE5I is recommended for men healthy enough to engage in sexual activity, the drugs themselves, after extensive scrutiny, have not been associated with adverse cardiovascular events [9].

The covariates were those recorded in the 18 to 12 month interval prior to the initial prescription for TT prescription or PDE5I. The pre-prescription interval was the one year prior to the initial prescription, the post-prescription interval was 90 days following the initial prescription, thus the prescription cohorts were restricted to those men with a minimum of 18 months enrollment prior to and three months after their initial prescription. In the post-prescription interval, patients were followed until a diagnosis of acute non-fatal myocardial infarction, refilled first prescription, or 90 days following initial prescription, whichever occurred first.

For those who did not refill their initial prescription, we analyzed an additional 90-day interval (91–180 days post-prescription), a time when this group likely had minimal use of these drugs. A refill and a subsequent prescription were treated equally in the analysis. Patients with first prescriptions for both TT prescription and PDE5I during follow-up were excluded from this analysis.

### Outcome

The study outcome was a diagnosis of acute MI (ICD-9: 410). Men with a history of MI prior to the first prescription for TT or PDE5I were excluded from the post-prescription analyses.

### Covariates

Age at the time of initial prescription was included as a covariate. Diagnostic covariates were identified by the ICD 9 codes recorded for inpatient or outpatient diagnoses, reported to be associated with MI, [10] including angina, arrhythmia, heart disease, prior MI, heart failure, hypertension, hyperlipidemia, stroke, peripheral vascular disease, cerebrovascular disease, transient ischemic attack, renal disease, obesity, asthma, chronic obstructive pulmonary disease, bronchitis, emphysema, alcohol-induced liver disease, alcohol dependence, and rheumatoid arthritis, osteoarthritis and arthritis NOS. Prescription covariates included use of anticoagulants, antiplatelet drugs, ACE inhibitors, glycosides, anti-arrhythmic drugs, alpha/beta blockers, beta-blockers, calcium channel blockers, hypolipidemic drugs, anti-hypertensive drugs, vasodilators, other cardiac drugs (includes 38 drugs each with very low prescription rates), non-steroidal anti-inflammatory drugs (NSAIDs), selective serotonin re-uptake inhibitors (SSRIs), corticosteroids, insulin, diuretics, and anti-diabetes drugs. All of these were included for analysis, but we restrict descriptive tabulations included in the manuscript to exposures that were ≥2%. Full descriptive tabulations are provided as supplementary material.

### Analyses

We examined risk of MI with TT prescription in all subjects. Since previous studies indicated that cardiovascular events appeared early in treatment, we focused on the 90 day interval following the filling of a first prescription. For those who did not refill their prescription, we also assessed the interval of 91–180 days. To examine potential effect modification by pre-existing disease, we estimated the effect of TT prescription by prior history of a heart disease diagnosis (ICD-9: 404, 414–414.07, 420–429). We also examined the RR for PDE5I and compared the RRs in the TT prescription and PDE5I cohorts.

### Statistical Methods

We examined the effect of the medications by estimating the ratio of the MI incidence rate in the post-prescription interval to the MI incidence rate in the pre-prescription interval (post/pre RR). To estimate the effect of TT prescription relative to PDE5I, we weighted the PDE5I patients with weights derived from propensity scores, specifically their estimated odds of being prescribed TT prescription rather than PDE5I. [11] This weighting aligns the distribution in the comparison cohort of the variables used in the prescription-probability model to match the distribution in the TT prescription cohort. These weights were then used in a Poisson regression model for the MI rate to obtain doubly robust estimates of effect. [12] These estimates are unconfounded by the adjustment variables if the prescription-odds model or the outcome-regression model is specified correctly. [13] To the extent that either model is approximately correct, any channeling bias due to the adjustment variables would be removed by this adjustment process. [13] In addition, we computed the weights so that they would result in equal pre-prescription MI incidence rates in the TT and PDE5I cohorts.

We also estimated the ratio of rate ratios (RRR). [13] The numerator of the RRR is the rate ratio for TT prescription relative to PDE5I in the post-prescription interval, and the denominator is the rate ratio for TT prescription relative to the PDE5I in the pre-prescription interval. This measure adjusts the post-prescription rate ratio for the corresponding rate ratio in the pre-prescription interval, and also controls for any differences in ascertainment between the pre- and post-prescription periods. It is intended to account for otherwise uncontrolled differences in the baseline rates of the cohorts. We estimated RRR from a Poisson regression model with MI as the outcome, log-exposure time as an offset, a drug indicator, and the patient features listed above. Since pre-prescription rates were weighted to be identical, the coefficient of the product of the indicator variables for the prescription type is the natural logarithm of RRR. All statistical analyses were conducted using the STARx and SAS software packages (STARx, CRI, Los Angeles, California, SAS 9.2, SAS Institute Inc., Cary, NC).

## Results

For all TT prescription subjects combined, the post/pre prescription rate ratio for MI (RR)was 1.36 (1.03, 1.81) (Table 1). In men aged 65 years and older the RR was 2.19 (1.27, 3.77), while in men under age 65 years the RR was 1.17 (0.84, 1.63). The difference in RR between men 65 and older and those under 65 reflects a broader trend of increasing RR with increasing age. The RRs were 0.95 (0.54, 1.67) under 55 years, 1.35 (0.77, 2.38) at 55–59, 1.29 (0.71, 2.35) at 60–64, 1.35 (0.44, 4.18) at 65–69, 1.62 (0.51, 5.16) at 70–74, and 3.43 (1.54, 7.66) at 75 years and older (p_trend_ = 0.03). Men aged 65 years and older, whose follow-up was not right-censored because they did not refill their prescription, were followed for an additional 90 day period (91–180 days post-prescription) during which the RR was 0.98 (0.43, 2.23). In men under age 65 the corresponding RR was 1.15 (0.79, 1.68).

**Table 1:** Rates of myocardial infarction per 1,000 persons per year (PY) in men under age 65 years and those age 65 years and older, in pre- and post-prescription intervals for an initial prescription for testosterone therapy rate ratios (RR) and 95% confidence intervals (CI).

	All Ages	Age <65 Years	Age ≥65 Years
Patients (N)	55,593	48,539	7,054
Pre-prescription			
Cases	193	156	37
Rate per 1,000 PY (95%CI)	3.48 (3.02, 4.01)	3.22 (2.75, 3.77)	5.27 (3.81, 7.27)
Post-prescription			
Cases	65	45	20
Rate per 1,000 PY (95%CI)	4.75 (3.72, 6.05)	3.76 (2.81, 5.04)	11.52 (7.43, 17.86)
Rate Ratio (post/pre) (95%CI)	1.36 (1.03, 1.81)	1.17 (0.84, 1.63)	2.19 (1.27, 3.77)

For the comparison group of PDE5I users, the baseline distributions of prior cardiovascular diagnoses, risk factors, and medication use were less common than in the TT prescription cohort, but after weighting, the distributions of covariates in each prescription cohort were nearly identical (Table 2). This procedure also resulted in the same adjusted pre-prescription rates of MI in both prescription groups: 3.48 per 1,000 person-years (PY). The adjusted post/pre RR for PDE5I across all ages was 1.08 (0.93, 1.24) (Table 3). When the data were stratified into ages 65 years and older and less than age 65 years, weighting also achieved nearly identical distributions of the covariates between the TT and PDE5I cohorts for each age group (Tables S1 and S2). In men aged 65 years and older, the adjusted RR for PDE5I was 1.15 (0.83, 1.59), and in those under age 65 years it was 1.06 (0.91, 1.24) (Table 3). The adjusted ratio of the rate ratios (RRRs) comparing those for TT prescription to those for PDE5I were 1.27 (0.94, 1.71) for all subjects, 1.90 (1.04, 3.49) for those aged 65 years and older and 1.10 (0.78, 1.56) for those under age 65.

**Table 2:** Distribution of baseline covariates for all Medicare and commercial insurance enrollees in the TT prescription and PDE5I cohorts before and after weighting.

Variable	TT Prescription	PDE5I Before Weighting	PDE5I After Weighting
N	55,593	167,279	141,031
Age, years	54.4	56.0	54.3
Medicare, %	12.3	14.5	12.0
Prior Diagnoses (ICD-9), %			
Hyperlipidemia (272)	26.5	23.5	26.3
Hypertension (401–405)	26.8	24.7	26.5
Heart Disease (404,414,420–429)	10.4	9.2	10.2
Osteoarthritis (715,720–721)	8.5	6.0	8.3
Asthma (493,495)	2.3	1.6	2.2
Prior Prescriptions, %			
Anticoagulant	2.8	2.4	2.7
Antiplatelets	3.4	2.8	3.3
Ace Inhibitors	18.1	18.8	18.3
Beta Blockers	15.5	15.2	15.6
Calcium Channel Blockers	11.7	11.4	11.8
Hypolipidemics	37.0	33.7	36.7
Anti-hypertensives NOS	3.2	3.1	3.2
Vasodialators	2.0	1.3	1.9
Other cardiac drugs	13.8	11.5	13.6
NSAIDs	16.3	13.5	16.1
SSRIs	20.7	11.8	20.4
Corticosteroids	12.7	9.2	12.6
Insulin	3.9	2.9	3.9
Diuretics	10.4	9.9	10.4
Anti-diabetes drugs	15.2	12.7	15.0

The TT prescription patients were unweighted and the PDE5I patients were weighted to match the TT prescription cohort based on odds of TT prescription.

These descriptive tabulations are restricted to exposures that occur in at least 2% or more of individuals. Please see the Tables S1 and S2 for a full list.

**Table 3:** Rates of myocardial infarction per 1,000 persons per year (PY) in men under age 65 years and those age 65 years and older, in pre- and post-prescription intervals for an initial prescription for PDE5I with adjusted* rate ratios (RR), and 95% confidence intervals (CI).

	All Ages	Age <65Years	Age ≥65Years
Patients (N)	167,279†	141,512†	25,767†
Pre-prescription			
Cases	695	556	139
Rate per 1,000 PY (95%CI)	3.48 (3.02, 4.01)	3.22 (2.75, 3.77)	5.27 (3.81, 7.27)
Post-prescription			
Cases	152	119	33
Rate per 1,000 PY (95%CI)	3.75 (3.19, 4.40)	3.42 (2.76, 4.24)	6.06 (4.26, 8.63)
Rate Ratio (post/pre) (95%CI)	1.08 (0.93, 1.24)	1.06 (0.91, 1.24)	1.15 (0.83, 1.59)

* Adjusted for age and pre-existing medical conditions and medication use associated with MI or its risk factors (Table 1 and Supplemental Tables).

† Effective sample sizes of PDE5I cohorts after weighting:

All Ages: 141,671.

Age <65: Years 121,696.

Age ≥65: Years 19,505.

The data for both prescription groups were also divided into men with a previously recorded diagnosis of any heart disease, and those without (Table 4). For TT prescription, in men under age 65 years, the RR was 2.90 (1.49, 5.62) for those with a history of heart disease and 0.90 (0.61, 1.34) for those without. In men aged 65 year and older, the RR was 2.16 (0.92, 5.10) for those with a history of heart disease and 2.21 (1.09, 4.45) for those without. The comparable RRs for PDE5I for those under age 65 years were 1.40 (0.91, 2.14) with a history of heart disease and 0.99 (0.84, 1.17) for those without. For PDE5I among those aged 65 years and older, the RR was 1.13 (0.68, 1.88) for men with a history of heart disease, and 0.92 (0.60, 1.39) for those without. The corresponding RRRs for TT prescription compared to PDE5I were 2.07 (1.05, 4.11) for those under age 65 years with a history of heart disease and 0.91 (0.60, 1.37) for those without, and 1.90 (0.66, 5.50) for those aged 65 years and older with a history of heart disease, and 2.41 (1.12, 5.17) for those without.

**Table 4:** Rates of myocardial infarction in men under and 65 and those 65 and older per 1,000(PY) in pre- and post-prescription intervals for an initial prescription for TT or PDE5 inhibitors, with adjusted* rate ratios (RR), ratio of rate ratios (RRR) and 95% confidence limits (CL) by history of heart disease.

	Heart Disease History		No Heart Disease History	
	TT Prescription	PDE5I	TT Prescription	PDE5I
Age <65 Years				
Patients (N)	4,006	10,681†	44,533	130,831†
Pre-prescription				
Cases	21	65	135	491
Rate per 1,000 PY (95%CI)	5.26 (3.43, 8.06)	5.26 (3.43, 8.06)	3.04 (2.57, 3.60)	3.04 (2.57, 3.60)
Post-prescription				
Cases	15	20	30	99
Rate per 1,000 PY (95%CI)	15.22 (9.18, 25.25)	7.34 (6.89, 7.82)	2.73 (1.91, 3.91)	3.01 (2.95, 3.08)
Rate Ratio (post/pre) (95%CI)	2.9 (1.49, 5.62)	1.4 (0.91, 2.14)	0.90 (0.61, 1.34)	0.99 (0.84, 1.17)
RRR‡ (95%CI)	2.07 (1.05, 4.11)		0.91 (0.60, 1.37)	
Age ≥65 Years				
Patients (N)	2,047	5,492†	5,057	20,275†
Pre-prescription				
Cases	15	35	22	104
Rate per 1,000 PY (95%CI)	7.36 (4.44, 12.22)	7.36 (4.44, 12.22)	4.41 (2.90, 6.7)	4.41 (2.90, 6.7)
Post-prescription				
Cases	8	13	12	20
Rate per 1,000 PY (95%CI)	15.91 (7.96, 31.81)	8.35 (7.36, 9.48)	9.74 (5.53, 17.14)	4.04 (3.69, 4.42)
Rate Ratio (post/pre) (95%CI)	2.16 (0.92, 5.10)	1.13 (0.68, 1.88)	2.21 (1.09, 4.46)	0.92 (0.60, 1.39)
RRR‡ (95%CI)	1.90 (0.66, 5.50)		2.41 (1.12, 5.17)	
Under 65 with heartdisease history	9,003			
Under 65 without a history of heart disease	112,588			
65 and older with heart disease history	4,190			
65 and older without a history ofheart disease	15,718			
‡ RRR = RR TT cohort/RR PDE5I cohort				

* Adjusted for age and pre-existing medical conditions and medication use associated with MI or its risk factors.

† Effective sample size of PDE5 inhibitor cohort after weighting.

Under 65 with heart disease history: 9,003.

Under 65 without a history of heart disease: 112,588.

65 and older with heart disease history: 4,190.

65 and older without a history of heart disease: 15,718.

‡ RRR = RR TT cohort/RR PDE5I cohort.

As odds-of-treatment weighting is less familiar than unweighted regression methods, we also examined the effect of controlling for the covariates using an unweighted Poisson regression. In subjects aged 65 years and older, the RRR was 2.27 (1.17, 4.43), about 20 percent higher than the weighted estimate of 1.90, and in those under age 65 years with a history of heart disease, the RRR was 2.33 (1.01, 5.35), about 13 percent higher than the weighted estimate of 2.07. The lower estimates suggest that weighting in the primary analysis helped reduce possible upward confounding.

## Discussion

Among men aged 65 years and older, we observed a two-fold increase in the risk of MI in the 90 days after filling an initial TT prescription, the risk declined to baseline in the 91 to 180 days after initial TT prescription, among those who did not refill their prescription. Since we censored follow-up at the first refill, and the supply for most prescriptions was 30 to 90 days, it is likely that there was little use of the medication in the 91 to 180 day post-prescription interval when the risk declined. Thus, the pattern of change in risk by supply of testosterone is consistent with an effect of the drug, and underscores the concerns raised by three recent studies in predominantly older men [4]–[6].

Among younger men with a history of heart disease, we observed a two to three-fold increased risk of MI in the 90 days following an initial TT prescription and no excess risk in younger men without such a history. Among older men, the two-fold increased risk was associated with TT prescription regardless of cardiovascular disease history, although this analysis was based on relatively small numbers of MI cases in each subgroup. More relevant perhaps is the rapid increase with age in the prevalence of diagnosed and undiagnosed coronary artery disease reported from autopsy studies, both overall and among accident victims, [14], [15] so that advanced age may be a more sensitive indicator of coronary disease prevalence than prior diagnoses. The recent study of TT within the VA healthcare system detected no change in the rate ratio for TT and coronary disease in the presence of existing coronary disease (“interaction” *P* = 0.41) [6]. However, since that study had less than 200 men with normal coronary arteries, they likely had insufficient statistical precision to address this question. Overall, our own findings appear consistent with a higher frequency of thrombotic events following TT prescription among men with more extensive coronary vascular disease.

Our findings are consistent with a recent meta-analysis of placebo-controlled randomized trials of testosterone therapy lasting 12 or more weeks among mainly older men, which reported that testosterone therapy increased the risk of adverse cardiovascular-related events (OR = 1.54, 95%CI:1.09, 2.18), as well as *serious* adverse cardiovascular-related events (OR = 1.61, 95%CI:1.01, 2.56) which included myocardial infarction along with other conditions. [5] This association appeared unrelated to average baseline testosterone level (p = 0.70) but varied by source of funding (p = 0.03), with a stronger summary effect in a meta-analysis of studies not funded by the pharmaceutical industry (OR = 2.06, 95%CI:1.34, 3.17) compared with studies funded by the pharmaceutical industry (OR = 0.89, 95%CI:0.50, 1.60). A majority of the included studies were of men whose serum androgen levels were deemed to be below normal physiological levels (hypogonadism).

Taken together, the evidence supports an association between testosterone therapy and risk of serious, adverse cardiovascular-related events–including non-fatal myocardial infarction–in men. However, there is some evidence that low *endogenous* testosterone levels may also be positively associated with cardiovascular events [16], [17]. But, as extensively reviewed by Xu et al. [5], effects of endogenous and exogenous testosterone may differ. Exogenous testosterone (TT) is associated with physiologic changes that predispose to clotting and thrombotic disorders including increased blood pressure [18], polycythemia [19], reductions in HDL cholesterol [18], [20], and hyperviscosity of the blood and platelet aggregation. [20]–[23]; TT also increases circulating estrogens [24], [25] which may play a role in the observed excess of adverse cardiovascular-related events, given that estrogen therapy has been associated with this excess in both men and women. [26]–[29] The mechanisms linking estrogens to thrombotic events may be related to markers of activated coagulation, decreased coagulation inhibitors, and activated protein C resistance [30].

Despite plausible biologic mechanisms linking TT prescription to an elevated risk of MI, our study has limitations related to use of a health-care database that did not include information on the serologic or diagnostic indications for treatment. It also identified only subjects with non-fatal MIs, typically representing about 75% of the total incidence, and was based on the diagnosis of an attending physician, rather than a structured evaluation as might occur in a randomized trial. However, the accuracy of an MI diagnosis is considered to be reliable in such databases, [31] and the established risk factors for MI apply to both fatal and non-fatal events. [32] We were also unable to examine whether this excess was related to indications such as level of serum testosterone or hypogonadism.

We addressed potential confounding from measured and unmeasured risk factors by using each treated group as its own control, comparing risk before versus after the start of medication use during the short time-frame of our study, and by controlling for differences in the prevalence of risk factors between the two treatment groups. In the prescription-odds weighted regressions, we found no association between PDE5I prescriptions and the risk of MI, suggesting that the TT prescription-related risk of MI is more likely a drug effect, rather than a result of behavioral or other factors associated with prescription.

Furthermore, there is no reason to suspect that physicians excluded high-risk individuals from TT prescription or monitored them more closely, since the hypothesis relating TT prescription to adverse cardiovascular events was not widely known during the study period. Indeed, the initial prescription for over 90 percent of the patients in our study occurred prior to publication of this potential concern, [4] and subsequent prescribing information and advertisements for these products have not referred to adverse cardiovascular risks.

Further study is needed to examine the risk of a variety of specific serious adverse cardiovascular events in relation to TT dose and duration, and to assess if the risks of TT vary by level of serum testosterone and presence or absence of hypogonadal disease. The observed excess MI risk in younger men with a history of heart disease is a particular public health concern, as about 10 percent of the men in our study under age 65 years with a TT prescription had a history of heart disease.

Given the rapidly increasing use of TT, the current results, along with other recent findings emphasize the urgency of the previous call for clinical trials adequately powered to assess the range of benefits and risks suggested for such therapy. Until that time clinicians might be well advised to include serious cardiovascular events in their discussions with patients of potential risks, particularly for men with existing cardiovascular disease.

## Supporting Information

Table S1 Distribution of baseline covariates in men 65 and older in the TTand PDE5 inhibitor cohorts before and after weighting. The TT patients were unweighted and the PDE5 inhibitor patients were weighted to match the TT cohort based on odds of TT prescription.
(DOC)

Table S2 Distribution of baseline covariates in men under 65 in the TT and PDE5 inhibitor cohorts before and after weighting. The TT patients were unweighted and the PDE5 inhibitor patients were weighted to match the TT cohort based on inverse prescription probability.
(DOC)
