# Supplementary material for: Increased Risk of Non-Fatal Myocardial Infarction Following Testosterone Therapy Prescription in Men
Source: PLoS One. 2014 Jan 29;9(1):e85805. doi: 10.1371/journal.pone.0085805 (PMC3905977; doi:10.1371/journal.pone.0085805)
Supplement: Table S1 — Distribution of baseline covariates in men 65 and older in the TTand PDE5 inhibitor cohorts before and after weighting. The TT patients were unweighted and the PDE5 inhibitor patients were weighted to match the TT cohort based on odds of TT prescription. (DOC) [file pone.0085805.s001.doc]

Table S1 . Distribution of baseline covariates in men 65 and older in the TTand PDE5 inhibitor cohorts before and after weighting. The TT patients were unweighted and the PDE5 inhibitor patients were weighted to match the TT cohort based on odds of TT prescription.

| Variable | TT Prescription | PDE5I  Before Weighting | PDE5I  After Weighting |
| --- | --- | --- | --- |
| N | 7054 | 25767 | 19505 |
| Medicare (%) | 92.8 | 91.5 | 92.6 |
| Age (years) | 72.3 | 71.2 | 72.2 |
| **Prior Diagnoses** (%) |  |  |  |
| Angina | 2.6 | 1.8 | 2.6 |
| Arrhythmia | 2.2 | 1.6 | 2.2 |
| Rheumatoid | 0.8 | 0.8 | 0.9 |
| Arthritis | 0.1 | 0.1 | 0.1 |
| Heart Failure | 4.1 | 2.0 | 3.8 |
| Hyperlipidemia | 25.9 | 24.0 | 25.8 |
| Hypertension | 36.0 | 32.0 | 35.5 |
| Heart disease | 29.0 | 21.3 | 28.4 |
| Osteoarthritis | 14.0 | 9.5 | 13.5 |
| Peripheral vascular disease | 3.8 | 2.0 | 3.5 |
| Renal disease | 3.3 | 1.8 | 3.0 |
| Obesity | 1.0 | 0.5 | 0.8 |
| Cardiac symptoms | 18.3 | 12.9 | 17.7 |
| Cardiovascular disease | 18.5 | 13.2 | 18.0 |
| Cerebrovascular disease | 5.4 | 3.7 | 5.3 |
| Transient ischemic attack | 1.0 | 0.7 | 1.0 |
| Asthma | 2.6 | 2.0 | 2.4 |
| COPD | 5.6 | 4.0 | 5.3 |
| Prior myocardial infarction | 1.9 | 1.0 | 1.6 |
| Bronchitis | 1.8 | 1.3 | 1.8 |
| Emphysema | 0.8 | 0.6 | 0.8 |
| Alcohol induced liver disease | 0.7 | 0.4 | 0.5 |
| Alcohol dependence | 0.2 | 0.1 | 0.2 |
| **Prior Prescriptions** (%) | |  |  |
| Anticoagulants | 8.9 | 6.3 | 8.6 |
| Antiplatelets | 9.8 | 7.0 | 9.5 |
| Ace Inhibitors | 27.8 | 26.3 | 28.1 |
| Gylcosides | 3.8 | 2.2 | 3.5 |
| Antiarrhythmics | 2.6 | 1.7 | 2.4 |
| Alpha-beta blockers | 0.6 | 0.4 | 0.4 |
| Beta Blockers | 31.1 | 26.8 | 31.3 |
| Calcium Channel Blockers | 20.3 | 18.7 | 20.5 |
| Hypolipidemics | 57.2 | 51.3 | 56.5 |
| Anti-hypertensives NOS | 8.8 | 7.6 | 8.6 |
| Vasodialators | 6.6 | 3.5 | 6.1 |
| Other cardiac drugs | 21.1 | 16.6 | 20.6 |
| NSAIDs | 18.1 | 15.1 | 17.6 |
| SSRIs | 20.9 | 10.3 | 20.3 |
| Corticosteroids | 15.3 | 11.2 | 15.0 |
| Insulin | 6.8 | 3.2 | 6.4 |
| Diuretics | 22.2 | 17.9 | 21.8 |
| Anti-diabetes drugs | 24.9 | 17.2 | 24.3 |
